# The Influence of Virtual Reality Glasses Use on the Quality of Life of Older Adults: Protocol for a Prospective, Longitudinal Quasi-Experimental Study

**DOI:** 10.2196/74298

**Published:** 2025-12-23

**Authors:** Dèlia Borda Fortuny, Sandra Rivas López, Sara Martínez-Torres, Cristina Rey-Reñones, Francisco M Martín-Luján

**Affiliations:** 1 Institut Catala de Salut, Primary Healthcare Centre, Valls, Spain; 2 ISAC Study Group, Primary Healthcare Research Support Unit Camp de Tarragona, Institut Universitari d'Investigació en Atenció Primària Jordi Gol, c/Riudoms 53-55, Reus, Catalunya, 43302, Spain, +34 977778515; 3 Department of Basic Medical Sciences and Department of Medicine and Surgery, Faculty of Medicine and Health Sciences, Universitat Rovira i Virgili, Reus, Catalunya, Spain; 4 TIC-AP Study Group, Primary Healthcare Research Support Unit Camp de Tarragona, Institut Universitari d'Investigació en Atenció Primària Jordi Gol, Reus, Catalunya, Spain; 5 Primary Healthcare Study Group, Institut d'Investigació Sanitària Pere Virgili, Tarragona, Spain

**Keywords:** virtual reality, quality of life, aged, primary health care, public health

## Abstract

**Background:**

Older adults are a rapidly growing demographic and often face social isolation and limited access to outdoor activities due to mobility issues, health conditions, and environmental barriers. These limitations can negatively impact their well-being, leading to reduced physical activity, cognitive decline, and emotional distress. Virtual reality (VR) technology offers a promising solution to bridge this gap by enabling access to immersive virtual environments, which may enhance the quality of life for residents in nursing homes.

**Objective:**

The aim of this study is to assess whether the use of VR glasses in nursing homes improves the quality of life of older adults by reducing the challenges they face in participating in recreational activities outside their care facilities.

**Methods:**

This study will adopt a prospective, longitudinal quasi-experimental design conducted in nursing homes within a basic health area of Catalonia, Spain. The intervention period will span 1 year. Participants will use VR glasses to interact with virtual environments, and their quality of life will be measured using the Rivas-Borda Quality of Life Scale, based on other validated scales.

**Results:**

Outcomes will focus on variations in quality-of-life scores before and after the intervention, as determined by the Rivas-Borda Scale. Statistical analysis will include detailed metrics such as sample size, confidence intervals, and *P* values to evaluate the intervention’s effectiveness.

**Conclusions:**

This research seeks to confirm that VR technology can be a valuable tool for enhancing the quality of life in older adults residing in nursing homes, addressing issues like social isolation and limited access to outdoor activities in an innovative and engaging way.

## Introduction

### Background

Quality of life (QoL) in older adults is a multidimensional concept that encompasses various crucial aspects of well-being and fulfillment as individuals age [1]. The global increase in the older adult population, projected to nearly double from 12% in 2015 to 22% in 2050, presents significant challenges for societies worldwide [2]. This demographic shift requires innovative approaches to maintain and enhance the QoL for older adults, particularly as they face unique physical, cognitive, and social challenges associated with aging.

Active aging has emerged as a holistic framework aimed at enhancing physical, cognitive, and emotional health, thereby improving the QoL for older adults. By promoting physical activity, social engagement, and healthy lifestyle choices, active aging effectively addresses the challenges associated with aging [3-6]. Recent research has highlighted the importance of multifaceted interventions that target various domains of well-being, including physical health, cognitive function, and social connectivity [7,8].

In recent years, emerging technologies—including robotics, virtual reality (VR), and smart textiles—have gained prominence for fostering healthy lifestyles among middle-aged and older adult populations. These innovative technologies play a crucial role in addressing issues such as social isolation and supporting active aging [9]. VR, in particular, has demonstrated considerable versatility and moderate cost, with applications spanning health domains including pain management, cognitive health, physical rehabilitation, and social well-being [10-15].

The potential of VR in health care for older adults has been extensively explored in recent literature. Studies have shown promising results in areas such as fall prevention, cognitive stimulation, and physical therapy [16,17]. Moreover, VR interventions have been found to improve mood and reduce anxiety in older adults, contributing to overall psychological well-being [18,19].

Despite these advancements, some studies have primarily focused on enhancing physical and cognitive functional capacities of individuals affected by specific diseases; a holistic perspective on QoL improvement remains underexplored. Current studies predominantly focus on neurodegenerative diseases and fall prevention in neurological patients, often overlooking broader determinants of QoL and their integration into aging interventions [20-22].

This study aims to assess whether 4 practical sessions, conducted using VR glasses, can improve QoL in older adults residing in geriatric facilities—a population frequently experiencing diminished well-being. Furthermore, this research emphasizes the importance of collaboration across various societal domains to address the multifaceted challenges of aging. Specifically, it integrates the social dimension, leveraging the support of the local municipal organization VallsGenera; the educational dimension, through partnerships with local schools; the technological dimension, using VR technology; and the health care dimension, by addressing the well-being and mental health of older adults in geriatric facilities. By fostering these interdisciplinary collaborations, this innovative approach aspires to bridge the digital divide faced by older adults and comprehensively enhance their QoL.

The integration of VR technology in geriatric care represents a promising avenue for enhancing the quality of life of older adults. By providing immersive experiences that stimulate cognitive function, promote physical activity, and facilitate social interaction, VR interventions have the potential to significantly impact various dimensions of well-being [8,19]. This study contributes to the growing body of research on technology-assisted interventions for older adults and aims to provide valuable insights into the efficacy of VR in improving overall quality of life in geriatric populations.

### Hypothesis

Participants who engage in the VR experience will report a significant improvement in perceived psychosocial well-being, as measured by the study-specific questionnaire.

## Methods

### Study Objectives

The general objective of this study is to evaluate whether conducting 4 practical VR sessions using VR glasses improves the quality of life of older adults.

The specific objectives are as follows:

- To analyze whether the emotional and cognitive well-being of participants improves according to the questionnaire created for this study.
- To identify whether there is an improvement in the sociofamiliar and physical dimension areas of the participants according to the questionnaire created for this study.
- To assess user satisfaction with practical VR sessions using VR glasses for older adults.

### Study Design, Setting, and Period

This is a prospective longitudinal quasi-experimental study designed to assess the impact of VR sessions on the QoL of older adults. The study will be conducted in all nursing homes within the basic health area of Valls (Catalonia, Spain). Valls is an inland city in the southern part of Catalonia and has a population of 25,047 [23].

**Figure 1.**
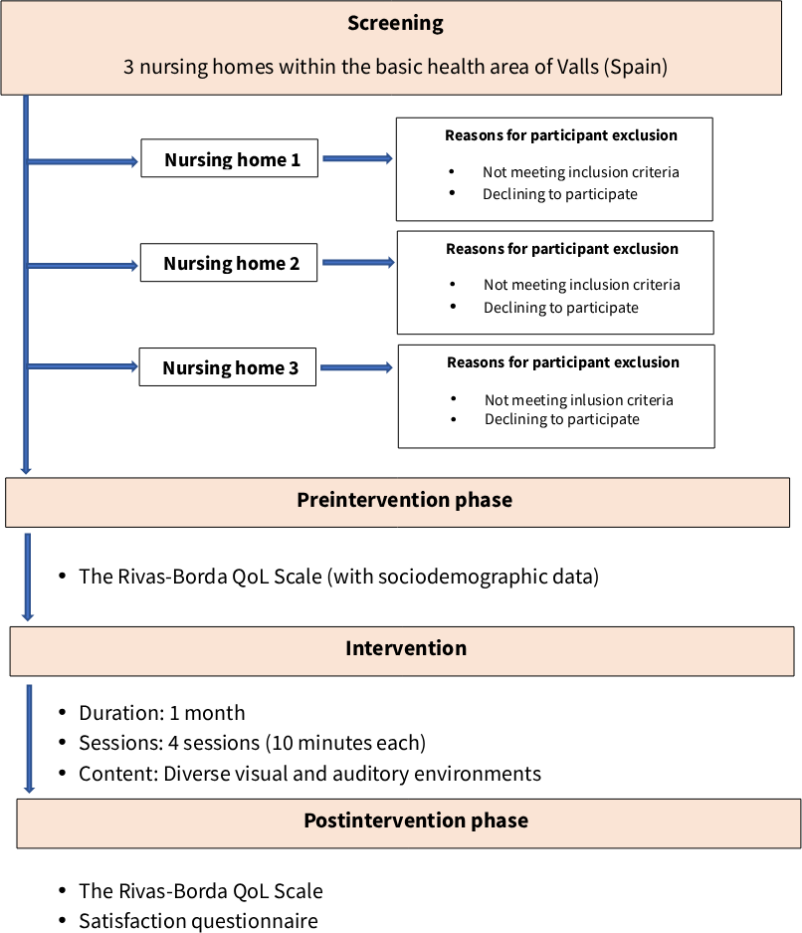
Flowchart of the VRLife study, showing the process of the screening, preintervention, intervention, and postintervention phases. QoL: quality of life.

The intervention period will span 1 year. The general framework and participant timeline of this study are shown in Figure 1.

### Institutional Involvement

The study involves collaboration with multiple entities: the Narcís Oller Institute, where students volunteered to assist with the intervention; VallsGenera, which will provide the necessary resources for the study; and all geriatric residences of Valls, named Alt Camp, Montserrat Cuadrada, and l’Alba, which will actively participate in the project.

Secondary school students from the Narcís Oller Institute, who were previously invited to participate, will visit the residential facilities to support the VR intervention. Their role has been clearly defined to focus primarily on technical assistance, with limited and standardized social interaction. Students will receive structured training from the nurses leading the study, which specifies the aspects of the intervention they may guide or assist with, ensuring consistency across sessions and minimizing variability in interpersonal interactions. The nurses will oversee all intervention procedures, provide supervision, and coordinate scheduling with both the educational institution and residential centers to arrange the sessions.

### Participant Selection

Eligible participants will include older adults residing in nursing homes in Valls who experience limitations in participating in recreational activities outside their care facilities. All residents who meet the criteria will be considered as potential participants. Invitations to participate will be extended in accordance with the predefined inclusion and exclusion criteria.

Inclusion criteria are as follows:

- Aged 65 years or older
- Individuals presenting with slight, moderate, significant, or complete impairment in social resources, as assessed by the Older Americans Resources and Services (OARS) methodology developed by Duke University (1978), which was adapted into a Spanish version [24].

Exclusion criteria are as follows:

- History of frequent migraines or motion sickness
- Moderate to severe cognitive impairment (≥5 errors on the Abbreviated Pfeiffer Test) [25]
- Advanced visual and/or hearing impairment
- Inability to communicate in either Spanish or Catalan

### Recruitment and Data Collection

The study will be presented to the management teams of the participating nursing homes. Upon approval, all residents will be informed about the study and invited to participate. Eligible individuals will be identified by the research team based on their residence in the basic health area of Valls and their compliance with inclusion and exclusion criteria. Those meeting the inclusion criteria and none of the exclusion criteria will be formally invited to participate.

Interested participants will receive detailed information about the study and will be asked to provide written informed consent prior to enrollment. As part of the screening process, participants will complete both the Abbreviated Pfeiffer Test and the OARS questionnaire, which will be used to assess cognitive status and social resources, respectively, in relation to the eligibility criteria.

### Sample Size

The effect size of VR on QoL has been established at 20 points [26]. Accepting an α risk of 0.05 and a power of 0.9 in a 2-tailed test, 59 subjects are needed in the observed group to recognize a difference as statistically significant. A dropout rate of 15% has been anticipated. The sample size was calculated using GRANMO (version 8) [27], a general, independent power analysis program for commonly used statistical tests in research.

### Description of the Intervention

Following the screening process, all eligible participants will undergo the intervention (Multimedia Appendix 1). The program consists of 4 VR sessions, each lasting 10 minutes, delivered once per week over a 1-month period. Sessions will take place in a safe, open space that allows for comfortable movement, with participants seated and supervised by trained nursing staff. Participants will use Conformité Européenne–marked NK VR headsets to access immersive 360° videos featuring passive, recreational content. These videos depict outdoor landscapes such as forests, beaches, mountains, and parks. A total of 10 distinct videos are available, and participants may choose freely among them and repeat any session if desired. The VR experience includes both visual and auditory elements to enhance the sense of presence and engagement. The intervention is standardized across all sessions and participants, ensuring consistency in delivery while allowing for individual preferences in video selection.

VR headsets will be disinfected between each use in accordance with institutional hygiene protocols to ensure participant safety and minimize the risk of cross-contamination.

To assess outcomes, participants will complete the World Health Organization Quality of Life of older adults (WHOQOL-OLD) questionnaire at 3 time points: baseline (preintervention), immediately postintervention, and at follow-up (3‐5 mo after the intervention). This validated instrument serves as the primary measure of QoL in this study.

In addition, participants will complete the Rivas-Borda QoL Scale, an exploratory tool developed ad hoc for this project. This scale assesses 4 dimensions of quality of life—sociofamilial, physical, emotional, and cognitive—using a 4-level frequency rating (never/almost never, sometimes, frequently, always/almost always). Although not yet formally validated, the scale is theoretically grounded in established instruments such as WHOQOL-OLD [28], EQ-5D [29], and the 36-Item Short Form Health Survey [30]. Detailed information about the Rivas-Borda Scale is provided in Multimedia Appendix 2.

#### Study Variables and Data Sources

The primary outcome will be the difference in QoL scores before and after the intervention, measured using the Rivas-Borda QoL Scale.

The criteria used to establish QoL will be the following:

- Adequate level of QoL: 81‐104 points
- Risk of QoL deterioration: 54‐80 points
- Impaired QoL: 23‐53 points

Independent variables included:

- Sociodemographic data included in the Rivas-Borda QoL Scale: gender (woman, man, or nonbinary), age (years), and marital status (single, widowed, married or with a partner living in the nursing home, married or with a partner not living in the nursing home)
- Satisfaction regarding the intervention, measured through a satisfaction questionnaire composed of a 3-question survey with 4 response options (Multimedia Appendix 3)
- Cognitive impairment, measured with the Pfeiffer Abbreviated Test
- Social resources, measured with the OARS

It is important to note that this information will be provided voluntarily by the older adult participants in the study and will be pseudonymized. Different variables are specifically detailed in Table S1 in Multimedia Appendix 4.

### Statistical Analysis

Quantitative data following a normal distribution will be presented as means and standard deviations, while nonnormally distributed quantitative data will be expressed as medians and interquartile ranges. The normality of variables will be assessed using the Kolmogorov-Smirnov test. Categorical data will be reported as frequencies and percentages.

First, a descriptive analysis of the study population will be conducted. Then, comparisons will be made to evaluate changes in variables before and after the intervention within the same group (paired analysis). For nonnormally distributed variables or sample sizes smaller than 30, the Wilcoxon signed-rank test will be used. In contrast, the paired Student *t* test will be applied to variables that follow a normal (Gaussian) distribution.

### Ethical Considerations

The study will be conducted in agreement with the principles of the Declaration of Helsinki and the guidelines of Good Clinical Practice. The Clinical Research Ethics Committee of the Primary Care Research Institute Jordi Gol (IDIAP Jordi Gol) (23/155P) approved this protocol. Data confidentiality will be protected by the Spanish law governing the protection of personal data (*Ley Orgánica de Protección de Datos de Carácter Personal y garantía de los derechos digitales*; 03/2018, of December 5). All participants will receive an information sheet from which they will be informed that their participation is anonymous, confidential, and voluntary and that they have the right to change their mind (not to participate) at any time up to data verification. Participants will be able to ask questions and receive clarification from the research team before deciding to participate. Verbal and written consent will be obtained from all participants to participate and be audio-recorded in the study. There will be no financial compensation for participation in the study.

All of the information from the study will be recorded consistently using a data collection questionnaire designed for this purpose. Each participant will be assigned a personal identification code upon inclusion. All information obtained will be stored using a digital application accessible only from the corporate intranet of the Catalan Health Institute in Tarragona. Access to this site is restricted and will be controlled by a personal password for each investigator, who will be responsible for data entry for all of the participants recruited. This data will not be publicly available, but the principal investigator may access it upon reasonable request and with the approval of the Research Ethics Committee for Human Subjects at IDIAP Jordi Gol. To disseminate the findings, a summary of the results will be emailed to the participants’ residential centers and presented to the research teams. The findings will also be shared with the scientific community through peer-reviewed publications and conference presentations, and dissemination in nonspecialized settings is also planned.

## Results

The study anticipates that VR interventions will significantly enhance the QoL of older adults, with expected improvements in cognitive engagement, emotional regulation, physical activity, and social participation [27]. Moreover, by introducing this technology, participants may gain confidence in interacting with digital tools, contributing to reduced technological apprehension and fostering broader digital inclusion.

This research has not received any specific grant funding from public, commercial, or nonprofit funding agencies as of December 2025. The research team will apply for specific funding opportunities. As of December 2025, the research team has conducted a pilot study with 22 participants. Preliminary data analysis has been completed, and the results are expected to be published during the first half of 2026.

## Discussion

### Rationale and Study Design

The study has been designed to address the often-overlooked aspect of QoL in older adults, emphasizing its importance across cognitive, physical, and emotional domains. By using VR technology, we seek to improve various dimensions of well-being, including cognitive, physical, and emotional domains. This approach aligns with recent research highlighting the potential benefits of VR in health care applications for older adults, such as cognitive stimulation, physical rehabilitation, and emotional well-being [31,32].

Furthermore, the research seeks to bridge the digital divide experienced by older adults by introducing and familiarizing them with new technologies, aiming to reduce the gap in digital literacy and promote a sense of empowerment and inclusion.

### Strengths

One of the key strengths of this study is its focus on QoL, a critical factor that is often neglected in older adults despite its profound influence on several dimensions of well-being including cognitive, physical, and emotional. By introducing VR technology in a supportive environment, we aim to increase digital literacy and access to technological benefits among this demographic [32].

The use of VR also provides a safe and controlled setting for older adults to engage in activities that might otherwise be challenging or impossible in their daily lives, such as travel or physical exercise [33].

Another strength of our approach is the potential for VR to positively enhance the mental health of older adults, particularly those with cognitive impairments, by eliciting positive affective states and enhancing emotional regulation [34]. This intervention could significantly improve health-related QoL in this population.

### Limitations

It is important to acknowledge the limitations of our study. First, the focus on a specific locality may limit the generalizability of our results to other geographic or cultural contexts. Additionally, there is a general lack of knowledge about the integration of VR in clinical contexts, which may pose challenges in the implementation and interpretation of results.

Previous research in this field has often been characterized by small sample sizes and controlled designs, with significant diversity in VR systems and content used across studies. This heterogeneity makes it difficult to draw direct comparisons between studies and underscores the need for more rigorous methodological approaches [35,36].

A significant challenge in implementing VR technology with older adults is the potential complexity of use, especially for those unfamiliar with such devices. Some participants may find it difficult to incorporate VR into their daily lives or to learn how to use it effectively [37]. This difficulty can lead to feelings of frustration or inadequacy, potentially impacting the overall experience and outcomes of the intervention.

A limitation of this study is the relatively short duration and low frequency of the intervention (four 10-minute VR sessions over one month). This intervention may not be sufficient to produce measurable or sustained improvements in multidimensional outcomes such as QoL. The decision to adopt this format was based on feasibility and safety considerations in an institutionalized older adult population, where tolerance and fatigue are relevant concerns. Nevertheless, future research should evaluate more intensive interventions, including multiple sessions per week over extended periods, as well as the inclusion of follow-up assessments (eg, 3‐5 months postintervention) to better capture potential long-term effects.

### Conclusion

This study aims to fill gaps in existing research by demonstrating the potential benefits of VR technology in improving QoL for older adults. The limitations identified highlight the importance of continued exploration and iterative development of VR tools. Efforts should also be directed toward ensuring accessibility and addressing usability challenges, thereby making these technologies truly inclusive and effective for this population.

## Supplementary material

10.2196/74298 Multimedia Appendix 1 RATE-XR information.

10.2196/74298 Multimedia Appendix 2 Rivas-Borda Quality of Life Scale.

10.2196/74298 Multimedia Appendix 3 Satisfaction questionnaire.

10.2196/74298 Multimedia Appendix 4 Table S1. Description of the variables and the source of information about the Rivas-Borda Quality of Life Scale.

## Abbreviations

OARS: Older Americans Resources and Services
QoL: quality of life
VR: virtual reality
WHOQoL-OLD: World Health Organization Quality of Life of older adults
