# Supplementary material for: The Influence of Virtual Reality Glasses Use on the Quality of Life of Older Adults: Protocol for a Prospective, Longitudinal Quasi-Experimental Study
Source: JMIR Res Protoc. 2025 Dec 23;14:e74298. doi: 10.2196/74298 (PMC12724481; doi:10.2196/74298)
Supplement: Multimedia Appendix 1 [file resprot-v14-e74298-s001.docx]

### 1. Content of the XR Intervention

The intervention consists of immersive experiences delivered through open-access content available on the YouTube platform. The selected videos are specifically designed for relaxation, reminiscence, and mild cognitive stimulation. Content is non-commercial and accessible, and it has previously been tested in older populations.

### 2. Hardware and Software

The intervention uses NK Glasses for Smartphone (model NK-G04-VR). These are standalone VR glasses that do not require PC tethering. Key features include 360° rotation, adjustable interpupillary and focal distance, and compatibility with smartphone-based VR content. The device is CE-marked and conforms to EU Directive 2014/30/EU. Supporting documentation (User Manual and CE Declaration of Conformity) is available.

### 3. Delivery and Setting

Sessions take place in a designated open room that allows for limited movement. The intervention is primarily intended to be conducted in a seated position, although standing is permitted if participants feel comfortable. Each session lasts approximately 10 minutes and is conducted individually.

### 4. Supervision and Facilitators

All sessions are supervised by researchers who are healthcare professionals, specifically nurses specialized in family and community nursing. Facilitators receive prior training on the correct use of the device, session procedures, and safety monitoring.

### 5. Safety and Risk Management

Participants are monitored at all times during the intervention. The seated position is encouraged to minimize fall risk, and safe boundaries within the room are maintained. Researchers document any incidents and assess participant comfort and well-being at the end of each session.

### 6. Usability and Participant Experience

Facilitators assist participants in fitting and adjusting the VR glasses. At the end of each session, participants provide feedback on usability and satisfaction. This feedback, together with incident reports, is used to assess feasibility and safety in this population.

### 7. Physical Movement

The intervention does not require physical movement. However, some participants may spontaneously perform controlled movements (e.g., stretching of the upper or lower limbs) depending on the content. Such movements are monitored and documented.

### 8. Outcomes and Data Collection

In addition to participant satisfaction, the intervention collects data on [socio-familiar, physical, emotional and cognitive]. Data collection procedures are standardized and reported transparently to facilitate replication.
