# Supplementary material for: The Influence of Virtual Reality Glasses Use on the Quality of Life of Older Adults: Protocol for a Prospective, Longitudinal Quasi-Experimental Study
Source: JMIR Res Protoc. 2025 Dec 23;14:e74298. doi: 10.2196/74298 (PMC12724481; doi:10.2196/74298)
Supplement: Multimedia Appendix 2 [file resprot-v14-e74298-s002.docx]

## Multimedia Appendix 1

**Rivas-Borda Quality of Life Scale**

Gender of subject ... (male/female/non-binary)

Age ............... (in years)

Marital status ............... (single / widowed / married or partner cohabiting with them in the nursing home / married or partner not cohabiting with them in the nursing home)

SOCIO-FAMILIAL DIMENSION

| **Questions** | **Never or almost** **never** | **Sometimes** | **Frequently** | **Always or almost always** |
| --- | --- | --- | --- | --- |
| They feel support from their closest family members. | **1** | **2** | **3** | **4** |
| They have satisfactory social relationships. | **1** | **2** | **3** | **4** |
| Lately they have been engaged in activities that motivate them. | **1** | **2** | **3** | **4** |
| They have access to the resources and services they consider necessary in their life. | **1** | **2** | **3** | **4** |
| They usually have concerns about the money they have at their disposal. | **4** | **3** | **2** | **1** |
| They are satisfied with their usual place of residence. | **1** | **2** | **3** | **4** |

PHYSICAL DIMENSION

| **Questions** | **Never**  **or almost never** | **Sometimes** | **Frequently** | **Always or**  **almost always** |
| --- | --- | --- | --- | --- |
| They consider themselves to be in good health. | **1** | **2** | **3** | **4** |
| They perceive mobility difficulties that prevent them from engaging in leisure activities. | **4** | **3** | **2** | **1** |
| They regularly experience pain that limits their daily life. | **4** | **3** | **2** | **1** |
| They consider that they take too much medication. | **4** | **3** | **2** | **1** |
| They are satisfied with their sex life. | **1** | **2** | **3** | **4** |
| They frequently leak urine or  faeces. | **4** | **3** | **2** | **1** |

EMOTIONAL DIMENSION

| **Questions** | **Never or almost**  **never** | **Sometimes** | **Frequently** | **Always or almost**  **always** |
| --- | --- | --- | --- | --- |
| They are satisfied with their current state of health. | **1** | **2** | **3** | **4** |
| They usually enjoy their life. | **1** | **2** | **3** | **4** |
| They consider that their life has meaning. | **1** | **2** | **3** | **4** |
| They usually feel that they have energy. | **1** | **2** | **3** | **4** |
| They feel useful in life. | **1** | **2** | **3** | **4** |
| They consider themselves good-looking. | **1** | **2** | **3** | **4** |
| They usually have positive feelings such as joy, happiness, hope, motivation etc. | **1** | **2** | **3** | **4** |
| They feel good when they think about their future. | **1** | **2** | **3** | **4** |
| They often feel accompanied. | **1** | **2** | **3** | **4** |
| They feel that they have time to  relax. | **1** | **2** | **3** | **4** |

COGNITIVE DIMENSION

| **Questions** | **Never or almost**  **never** | **Sometimes** | **Frequently** | **Always or almost**  **always** |
| --- | --- | --- | --- | --- |
| They consider that they are well rested. | **1** | **2** | **3** | **4** |
| They tend to make their own decisions. | **1** | **2** | **3** | **4** |
| They think their memory is good. | **1** | **2** | **3** | **4** |
| They consider themselves autonomous in life. | **1** | **2** | **3** | **4** |
| They consider that they have a good quality of life. | **1** | **2** | **3** | **4** |

# Scoring:

- Adequate quality of life: 81-104 points.
- Risk of deterioration in quality of life: 54-80 points.
- Altered quality of life: 23-53 points.

## Multimedia Appendix 2

**Satisfaction questionnaire**

| **Questions** | **None** | **A little** | **Quite a lot** | **A lot** |
| --- | --- | --- | --- | --- |
| Do you consider it useful to have done this activity? | None | A little | Quite a lot | A lot |
| Would you recommend this activity to  someone else? | None | A little | Quite a lot | A lot |
| Would you like to do more sessions? | None | A little | Quite a lot | A lot |
