# Supplementary material for: The Influence of Virtual Reality Glasses Use on the Quality of Life of Older Adults: Protocol for a Prospective, Longitudinal Quasi-Experimental Study
Source: JMIR Res Protoc. 2025 Dec 23;14:e74298. doi: 10.2196/74298 (PMC12724481; doi:10.2196/74298)
Supplement: Multimedia Appendix 3 [file resprot-v14-e74298-s003.docx]

## Multimedia Appendix 2

**Satisfaction questionnaire**

| **Questions** | **None** | **A little** | **Quite a lot** | **A lot** |
| --- | --- | --- | --- | --- |
| Do you consider it useful to have done this activity? | None | A little | Quite a lot | A lot |
| Would you recommend this activity to  someone else? | None | A little | Quite a lot | A lot |
| Would you like to do more sessions? | None | A little | Quite a lot | A lot |
