# Supplementary material for: The Influence of Virtual Reality Glasses Use on the Quality of Life of Older Adults: Protocol for a Prospective, Longitudinal Quasi-Experimental Study
Source: JMIR Res Protoc. 2025 Dec 23;14:e74298. doi: 10.2196/74298 (PMC12724481; doi:10.2196/74298)
Supplement: Multimedia Appendix 4 [file resprot-v14-e74298-s004.docx]

Table S1. Description of the variables and the source of information

| **Variables** | **Screening** | **Source of information** | **Pre-intervention** | **Post-intervention** |
| --- | --- | --- | --- | --- |
| **Cognitive impairment** | Pfeiffer Abbreviated test |  |  |  |
| **Social Resources** | OARS Scale |  |  |  |
| **Sociodemographic data:** gender, years, marital status |  | Rivas-Borda QoL Scale | X |  |
| **Quality of Life:**   - Socio-familiar dimension - Physical dimension - Emotional dimension - Cognitive dimension |  | Rivas-Borda QoL Scale | X | X |
| **Satisfaction of the intervention** |  | Satisfaction questionnaire |  | X |
